# Unusual malar rash with ulceration: What can we expect?

**DOI:** 10.1002/ccr3.7163

**Published:** 2023-04-02

**Authors:** Kouki Chaima, Linda Manaa, Emna Bahloul, Sonia Boudaya, Mariem Amouri, Hamida Turki

**Affiliations:** ^1^ Department of Dermatology Hedi Chaker Hospital Sfax Tunisia

**Keywords:** cutaneous leishmaniasis, dermoscopy, erysiploid, parasitology

## Abstract

Considering the clinical polymorphism of the disease, longstanding skin lesions located on the face, resembling erysipelas in an endemic area should always be investigated for CL and thus, atypical presentations should be kept in mind.

## Graphical abstract

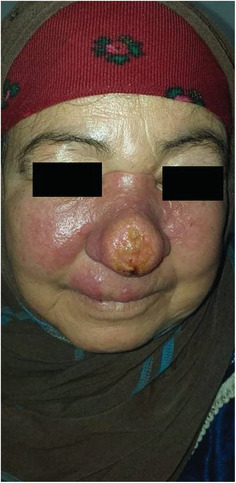

A 45‐year‐old Tunisian woman, with no medical history, presented with an infiltrative plaque of the face treated with a systemic antibiotic for 1 month with no significant response. Cutaneous examination revealed an erythematous infiltrate plaque covering the center of the face with an ulcerated center and extension to the cheeks. (Figure 1) The review of the underlying mucosa was normal. Dermoscopy showed multiple orange‐red areas with diffuse erythema, linear vessels, and multiple yellow tears. (Figure 2).

**FIGURE 1:**
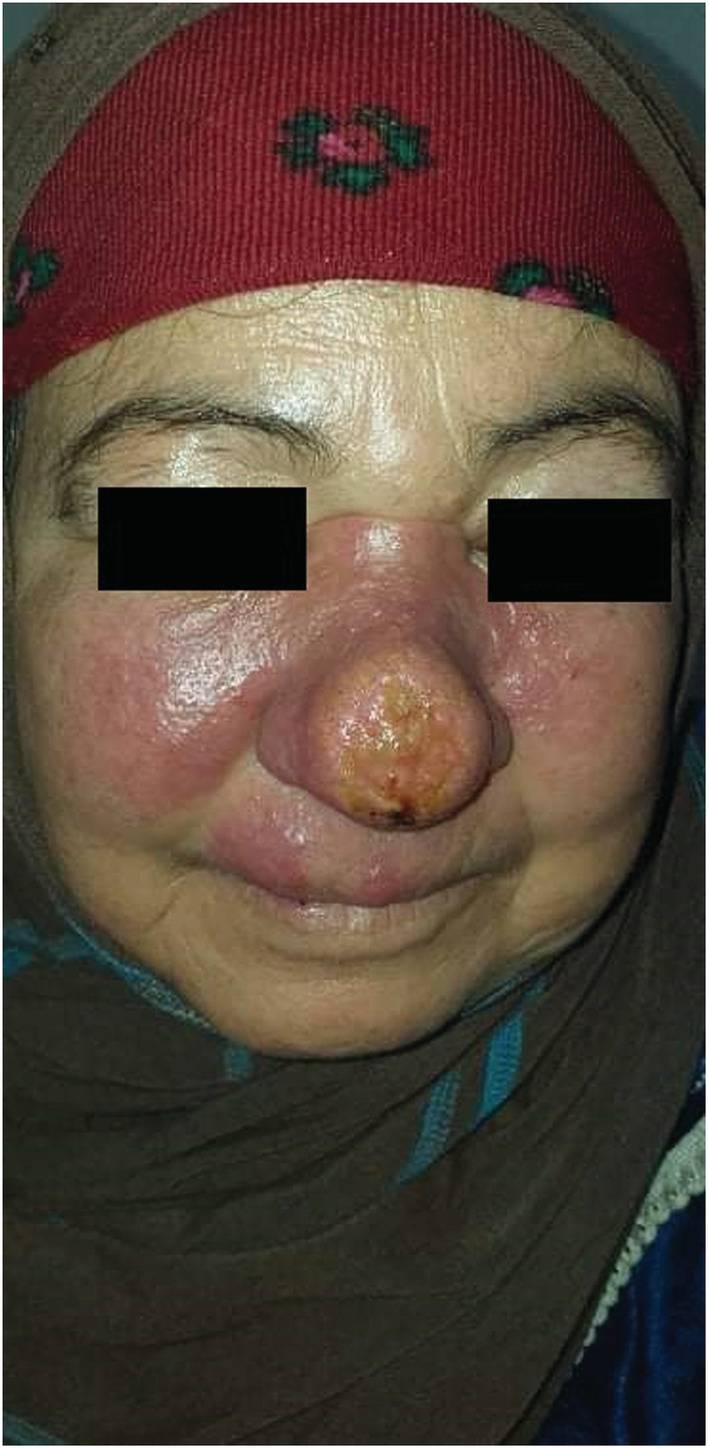
Erythematous infiltrate plaque covering the center of the face with an ulcerated center and extension to the cheeks.

**FIGURE 2:**
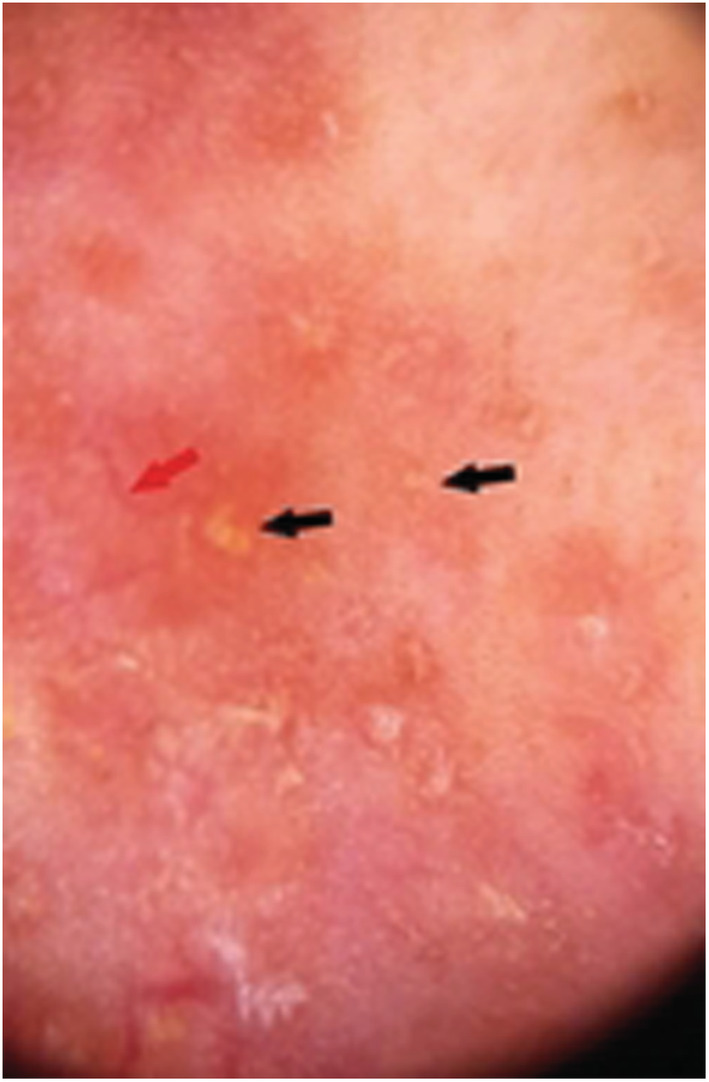
Dermoscopy: orange‐red areas and erythema, linear vessels (red arrows), and multiple yellow For Review Only tears (black arrows).

Given the endemo‐epidemic profile of our country, cutaneous leishmaniasis (CL) was suspected. Further evaluation by direct examination of the skin of the nose using lesion scraping by a vaccinostyle, under sterile conditions was performed (Giemsa staining). The smears detected intra and extracellular amastigote forms of Leishmania confirming the diagnosis of CL. The patient was treated with intramuscular meglumine antimoniate for 14 days with good evolution.

CL is a parasitic infection encountered in daily dermatologic practice. It has many different clinical presentations depending on the immune status of the host and subspecies of *Leishmania*.^1^ Erysipeloid CL is atypical and under‐reported of leishmaniasis. It frequency in the literature is estimated between 0.005 and 3.2%.^1^ Several hypotheses have been suggested: hormonal disturbances associated with menopause, alteration of immunity and the quality of skin tissue linked to aging, and specific species involved.^1^ Recently, multiple dermoscopic features of CL have been described including erythema, yellow tears, starburst‐like patterns, and vascular structures.^2^

## AUTHOR CONTRIBUTIONS

**kouki chaima:** Data curation; formal analysis; validation; visualization; writing – original draft; writing – review and editing. **Linda Manaa:** Formal analysis; methodology; writing – original draft; writing – review and editing. **emna bahloul:** Formal analysis; funding acquisition; supervision; validation; writing – original draft; writing – review and editing. **Sonia Boudaya:** Formal analysis; visualization; writing – original draft; writing – review and editing. **Mariem Amouri:** Conceptualization; methodology; validation; writing – original draft; writing – review and editing. **Hamida Turki:** Conceptualization; writing – original draft; writing – review and editing.

## FUNDING INFORMATION

We received no funding to support this work.

## CONFLICT OF INTEREST STATEMENT

We have no conflict of interest to disclose concerning this work.

## INFORMED CONSENT

Written informed consent was obtained from the patient to publish this report in accordance with the journal's patient consent policy.

## Data Availability

The data that support the findings of this study are available from the corresponding author upon reasonable request.
